# Dibenzoylmethane Exerts Metabolic Activity through Regulation of AMP-Activated Protein Kinase (AMPK)-Mediated Glucose Uptake and Adipogenesis Pathways

**DOI:** 10.1371/journal.pone.0120104

**Published:** 2015-03-10

**Authors:** Nami Kim, Hong Min Kim, Eun Soo Lee, Jung Ok Lee, Hye Jeong Lee, Soo Kyung Lee, Ji Wook Moon, Ji Hae Kim, Joong Kwan Kim, Su Jin Kim, Sun Hwa Park, Choon Hee Chung, Hyeon Soo Kim

**Affiliations:** 1 Department of Anatomy, Korea University College of Medicine, Seoul 136-701, Korea; 2 Department of Internal Medicine, Yonsei University Wonju College of Medicine, Wonju 220-701, South Korea; Tohoku University, JAPAN

## Abstract

Dibenzoylmethane (DBM) has been shown to exert a variety of beneficial effects on human health. However, the mechanism of action is poorly understood. In this study, DBM increased phosphorylation of AMP-activated protein kinase (AMPK) and stimulated glucose uptake in a skeletal muscle cell line. Both knockdown of AMPK with siRNA and inhibition with AMPK inhibitor blocked DBM-induced glucose uptake. DBM increased the concentration of intracellular calcium and glucose uptake due to DBM was abolished by STO-609 (a calcium/calmodulin-dependent protein kinase inhibitor). DBM stimulated phosphorylation of p38 mitogen-activated protein kinase (p38 MAPK), which was blocked by pretreatment with compound C, an AMPK inhibitor. The expression of glucose transporter type 4 (GLUT4) was increased by DBM. The translocation of GLUT4 to the plasma membrane was also increased by DBM in AMPK dependently. In addition, DBM suppressed weight gain and prevented fat accumulation in the liver and abdomen in mice fed a high-fat diet. In pre-adipocyte cells, DBM decreased the activity of acetyl-CoA carboxylase (ACC), the rate-limiting enzyme of fatty acid synthesis. Expression of the adipogenic gene, fatty acid synthase (FAS), was suppressed by DBM in an AMPK-dependent manner. These results showed that the beneficial metabolic effects of DBM might be due to regulation of glucose uptake via AMPK in skeletal muscle and inhibition of adipogenesis in pre-adipocytes.

## Introduction

The AMPK complex is an evolutionarily conserved sensor of cellular energy status [1, 2]. AMPK is activated in response to an increase in the cellular AMP: ATP ratio, which generally occurs as ATP levels decline [3–4]. Once activated, AMPK activates on ATP-generating pathways, and switches off ATP-consuming pathways. AMPK can also be activated via multiple regulatory pathways [5–6]. Although the molecular mechanisms underlying AMPK activation have not been thoroughly elucidated, it has previously been demonstrated that AMPK activation requires phosphorylation of the catalytic α subunit on threonine 172 within the activation loop [7–8]. Liver kinase B1 (LKB1) and calcium/calmodulin-dependent protein kinase (CaMKK) have also been associated with AMPK [9–12]. Upon activation, AMPK causes an increase in glucose uptake, fatty acid oxidation, and mitochondrial biogenesis.

Dibenzyolmethane (DBM; 1,3-diphenyl-propanedion) is a natural phytochemical found as a minor constituent of licorice [13] and is a β-diketone analogue of curcumin, the yellow pigment in the spice, turmeric. A variety of biological properties has been reported for DBM, including anti-inflammatory activity [14], anti-mutagenesis [15], cell cycle regulation [16], and anti-estrogenic activity [17]. We previously reported that curcumin stimulated glucose uptake through AMPK in skeletal muscle [18]. AMPK regulates a wide array of physiological events that have been linked to insulin resistance, which suggests that activation of AMPK by DBM may be a functional target for insulin resistance and related pathophysiological syndromes, such as diabetes and obesity. Curcumin analogues have been suggested as potential cancer drug treatments [19–21] and have shown potential in treating diabetes [22–23]. Despite these implications on the function of curcumin analogues, the mechanism by which DBM mediates on metabolism is yet to be established.

In order to characterize the metabolic effects of DBM more precisely, we conducted an investigation into the effects of DBM in a model of high-fat diet (HFD)-induced obesity. In this study, we found that DBM downregulates blood glucose levels and suppresses fat accumulation in this obesity model. We also showed that DBM exerts its role in glucose regulation via AMPK in a skeletal muscle cell line and via fatty acid synthase (FAS) suppression in a pre-adipocyte cell line. Together, our data suggest that DBM may affect metabolic processes both in L6 myotube cells and in 3T3-L1 adipocytes via AMPK activation.

## Materials and Methods

### Reagents

STO-609, a CaMKK inhibitor, and metformin were purchased from Sigma Chemical Company (St. Louis, MO, USA). 5-Aminoimidazole-4-carboxamide-1-β-ribofuranoside (AICAR) was purchased from Toronto Research Chemical Incorporation (Toronto, ON, Canada). DBM (1, 7-bis [4-hydroxy-3-methoxyphenyl]-1, 6-heptadiene-3, 5-dione), SB203580, a p38 MAPK inhibitor, was obtained from Biomol International LP (Butler Pike, PA, USA). Polyclonal anti-phosphorylated AMPKα, phosphorylated ACC, and phosphorylated p38 MAPK antibodies, and anti-AMPKα, ACC, p38 MAPK, β-actin antibodies were purchased from Millipore (MA, USA). Compound C, an AMPK inhibitor, was provided by Merck (RY 70–100; Rahway, NJ, USA). Hybond ECL nitrocellulose membranes were obtained from Amersham (Arlington Heights, IL, USA).

### Cell culture

Mouse myoblast C2C12 cells, rat myoblast L6 cells, and pre-adipocyte 3T3-L1 cells were maintained in Dulbecco’s modified Eagle medium (DMEM) supplemented with 10% heat-inactivated fetal bovine serum (FBS) and 1% antibiotics (100 U/mL penicillin and 100 μg/mL streptomycin) at 37°C in a humidified atmosphere with 5% CO_2_. For differentiation into myotubes to be used in glucose uptake studies, rat myoblast L6 cells were reseeded into 12-well plates at a density of 2 × 10^4^ cells/mL. After 24 h (at >80% confluence), the medium was changed to DMEM containing 2% (v/v) FBS and was replaced after 2, 4, and 6 days of culture. Experiments were initiated after 7 days when myotube differentiation was complete.

### Western blot analysis

C2C12, L6, or 3T3-L1 cells were grown in 6-well plates until they reached 60–70% confluence. Then the cells were subjected to 24 h of serum starvation prior to treatment at 37°C with selected agents. DBM was treated with concentration of 30 μM for the indicated times. Following treatment the medium was aspirated, and cells were washed twice in ice-cold phosphate-buffered saline (PBS), then lysed in 100 μL of lysis buffer (0.5% deoxycholate, 0.1% sodium dodecyl sulfate [SDS], 1% Nonidet P-40, 150 mM NaCl, and 50 mM Tris-HCl, [pH 8.0]) containing proteinase inhibitors (0.5 μM aprotinin, 1 μM phenylmethylsulfonyl fluoride, and 1 μM leupeptin) (Sigma). The supernatants were sonicated briefly, heated for 5 min at 95°C, centrifuged for 5 min, separated on SDS-polyacrylamide gel electrophoresis (8–16%) gels, and finally transferred to polyvinylidene difluoride membranes. The membranes were then incubated overnight at 4°C with primary antibodies, and washed 6 times in Tris-buffered saline with 0.1% Tween 20. Then, the membranes were incubated with horseradish peroxidase (HRP)-conjugated secondary antibodies for 1 h at room temperature. Anti-glyceraldehyde-3-phosphate dehydrogenase (GAPDH) antibodies were used to normalize protein loading. The blots were then visualized with an ECL Gel Electrophoresis System (Amersham Biosciences; Buckinghamshire, UK). The membrane was scanned and densitometry analysis was performed with an Image J analysis. Each experiment was repeated three times.

### RT-PCR

C2C12 or 3T3-L1 cells (1 × 10^6^ cells/ml) were treated with DBM (30 μM) or insulin (100 nM) for indicated times. Total RNA was isolated using the TRI Reagent (Life Technologies; Paisley, UK) following the manufacturer’s instructions. First-strand cDNA synthesis was performed using 1 μg of total RNA isolated from C2C12 cells at 55°C for 20 min using the Thermoscript II one-step RT-PCR Kit (Life Technologies; Paisley, UK). cDNA amplification was performed in the same tube using the Gene Amp System 9700 thermocycler (Applied Biosystems; Warrington, UK) followed by heating to 94°C for 5 min to inactivate reverse transcriptase. The following PCR conditions were used: 34 cycles each of 30 s at 94°C, 30 s at 55°C, and 60 s at 72°C, followed by 10 min at 72°C. The number of PCR cycles used was optimized to ensure amplification in the exponential phase. Ten-microliter samples from each RT-PCR reaction were removed and analyzed with agarose gel electrophoresis. Bands were stained with ethidium bromide and visualized under ultraviolet light. The band intensities were quantified using a gel documentation system (Gene Genius; Syngene, UK). The following primers were used: GLUT4-sense (5′-TTG GAG AGA GAG CGT CCA AT-3′) and GLUT4-antisense (5′-CTC AAA GAA GGC CAC AAA GC-3′), FAS-sense (5′-CAC ACA CAA TGG ACC CCC AG-3′) and FAS-antisense (5′-CAG AGG TGT TCG GCT TCA GG-3′), C/EBPα-sense (5′-AGG TGC TGG AGT TGA CCA GT-3′) and C/EBPα-antisense (5′-CAG CCT AGA GAT CCA GCG AC-3′), C/EBPβ-sense (5′-CGG GGT TGT TGA TGT TTT TGG-3′) and C/EBPβ-antisense (5′-TCG AAA CGG AAA AGG TTC TCA-3′), C/EBPdelta-sense (5′-CTT CGC CGA CCT CTT CAA C-3′) and C/EBPdelta-antisense (5′-CGC ACA CCG CCA CTT G-3′), aP2-sense (5′-AAG ACA GCT CCT CCT CGA AGG TT-3′) and aP2-antisense (5′-TGA CCA AAT CCC CAT TTA CGC-3′), SCD1-sense (5′-CCT TCT TGC GAT ACA CTC TGG TG-3′) and SCD1-antisense (5′-TCA GCT ACT CTT GTG ACT CCC G-3′), PPAR-gamma-sense (5′-GGT GAA ACT CTG GGA GAT TC-3′) and PPAR-gamma-antisense (5′-CAA CCA TTG GGT CAG CTC TT-3′), and β-actin-sense (5′-CAG GAG GAG CAA TGA TCT TGA-3′) and β-actin-antisense (5′-ACT ACC TCA TGA AGA TCC TCA-3′). Each experiment was repeated three times.

### Intracellular calcium measurement

Ca^2+^ concentration was determined by detecting fluorescence in C2C12 cells treated with the Ca^2+^-sensitive indicator, fluo-3 AM, using confocal microscopy (Zeiss LSM 510 Meta; Zeiss; Oberkochen, Germany). To measurement of intracellular calcium, C2C12 cells (1 × 10^4^ cells/ml) were seeded in 35 mm dishes. The cells were loaded with 5 mM fluo-3 AM in regular culture medium for 45 min at room temperature. The cells were washed, then incubated for 15 min in the absence of fluo-3 AM in order to de-esterify the dye. The culture plates were placed on a temperature-controlled microscope stage and viewed using the 20× objective. The cells treated with 30 μM of DBM, and then observed under confocal microscopy. The excitation and emission wavelength for signal detection was 488 nm.

2-Deoxy-D [H^3^] glucose (2-DG) uptake

L6 muscle cells were differentiated with DMEM supplemented with 2% FBS for 7 days. Glucose uptake was analyzed by measuring the uptake of 2-deoxy-D [H^3^] glucose (2-DG) in differentiated L6 myotubes. Cells were rinsed twice with warm PBS (37°C), and then starved in serum-free DMEM for 3 h. After pretreated with inhibitors for 30 min, cells were then treated with DBM (30 μM) for the indicated times. The cells were incubated in Krebs Ringer Henseleit (KRH) buffer (20 mM HEPES, pH 7.4, 130 mM NaCl, 1.4 mM KCl, 1 mM CaCl_2_, 1.2 mM MgSO_4_, and 1.2 mM KH_2_PO_4_) containing 0.5 μCi of 2-DG for 15 min at 37°C. The reaction was terminated by placing the plates on ice and washing them twice with ice-cold PBS. The cells were lysed in 0.5 N NaOH and 400 μL of the lysate and mixed with 3.5 mL of scintillation cocktail. Radioactivity was determined by scintillation counting.

### AMPKα2 silencing

Rat myoblast L6 cells were seeded in 6-well plates and allowed to grow to 70% confluence for 24 h. Transient transfections were performed with a transfection reagent, Lipofectamine 2000 (Invitrogen; Carlsbad, CA, USA) according to the manufacturer’s protocol. Briefly, AMPKα2 siRNA was purchased from Dharmacon (Thermo Scientific; L-040809-00-0005) and non-targeted control siRNA was designed and synthesized (Bioneer; Daejon, Korea). siRNA 5 μL and 5 μL of Lipofectamine 2000 were diluted with 95 μL of reduced serum medium (Opti-MEM; Invitrogen) and mixed. The mixture was incubated for 30 min at room temperature and added drop-wise to each culture well containing 800 μL of Opti-MEM (final siRNA concentration, 100 nM). Four hours after transfection, the medium was replaced with fresh complete medium for 48 h. After transfection with siRNA, DBM (30 μM) were treated for indicated times.

### Myc-GLUT4 translocation assay

Cell surface expression of GLUT4myc was quantified using an antibody-coupled colorimetric absorbance assay as previously described [24]. DBM of 30 μM was treated for 1 h. Following stimulation, myoblasts stably expressing L6-GLUT4myc were incubated with polyclonal anti-myc antibody (1:1000) for 60 min, fixed with 4% paraformaldehyde in PBS for 10 min, and incubated with HRP-conjugated goat anti-rabbit IgG (1:1000) for 1 h. Cells were washed 6 times in PBS and incubated in 1 mL of OPD reagent (0.4 mg/mL) for 30 min. The absorbance of the supernatant was measured at 492 nm.

### High fat diet in mice

Four-week-old male C57BL/6 mice were purchased from Dae Han Bio Link Co. (Chungbuk, Korea). The Korea University Institute Animal Care and Use Committee approved all of experimental procedures performed with animals. In addition, all experiments were performed in accordance with the guidelines of Korea University. They were housed in cages and placed in a room with a 12:12-h light-dark cycle and ambient temperature. The mice were fed a commercial diet for 4 weeks after arrival for adaptation. At 8 weeks of age, they were divided randomly into three groups with 10 animals per group. The groups were then fed a HFD, a HFD with DBM (100 mg/kg), or a normal diet for 12 weeks. Food intake and body weights were recorded weekly. After 12 weeks, animals were anaesthetized with Zoletil (Virvac Laboratories; Carros, France) by intraperitoneal injection.

### Data analysis

We used one-way ANOVA and Holm-Sidak comparisons and the post-hoc Fisher test to compare the potency of insulin secretion. The difference between the mean values was considered to be statistically significant when p < 0.05.

## Results

### DBM increased phosphorylation of AMPK in skeletal muscle cells in a time- and concentration-dependent manner

**Fig 1:**
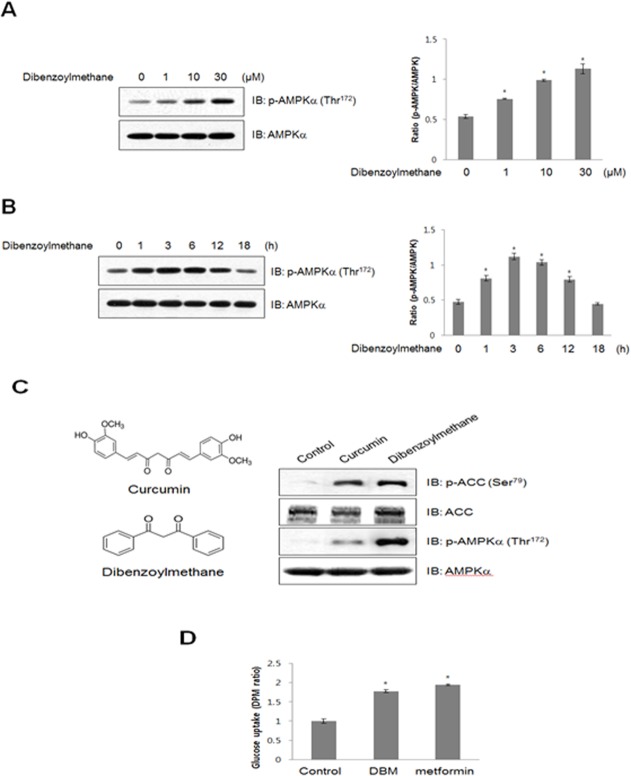
(A) C2C12 cells were stimulated for 1 h with various concentrations of DBM. The cells were then lysed with lysis buffer, and the phosphorylation of AMPKα was assessed by western blot using antibodies specific for the phosphorylated protein. The level of total AMPKα was also assessed as a control for protein loading. (B) C2C12 cells were treated with 30 μM DBM for the times indicated. The cells were lysed with lysis buffer, and the phosphorylation of AMPKα was evaluated by western blot using antibodies specific for the phosphorylated protein. The level of total AMPKα was also assessed as a control for protein loading. (C) C2C12 cells were treated with DBM and curcumin for 1 h. The cells were lysed with lysis buffer, and the phosphorylation of AMPKα and ACC was evaluated using antibodies specific for the phosphorylated protein. The level of total AMPKα and ACC was also assessed as a control for protein loading. (D) L6 myotubes were differentiated for 7 days and then treated with 30 μM DBM and 2 mM metformin for 18 h. 2-deoxy-D [H^3^] glucose (2-DG) uptake was then assayed, as described in the Methods. * p < 0.05, as compared with basal condition. The results presented are representative of three individual experiments.

To determine the mechanism underlying the metabolic effects of DBM in C2C12 myoblasts, we assessed the activation of AMPK, a key regulator of glucose uptake. The administration of DBM induced a concentration- and time-dependent increase in AMPK phosphorylation (Fig. 1A, B). Maximal AMPK phosphorylation was observed with a concentration of 30 μM DBM. The phosphorylation of AMPK reached a maximum at 3 h and then returned to basal levels at 18 h. DBM, analogue of curcumin, showed a stronger activity for the phosphorylation of AMPK and its downstream ACC than curcumin (Fig. 1C). Comparing with the potency for glucose uptake, DBM showed a similar activity with metformin, a known AMPK activator (Fig. 1D). These results indicate that DBM increased the phosphorylation of AMPK in a skeletal muscle C2C12 cells.

### DBM increased 2-DG uptake in an AMPK-dependent manner

**Fig 2:**
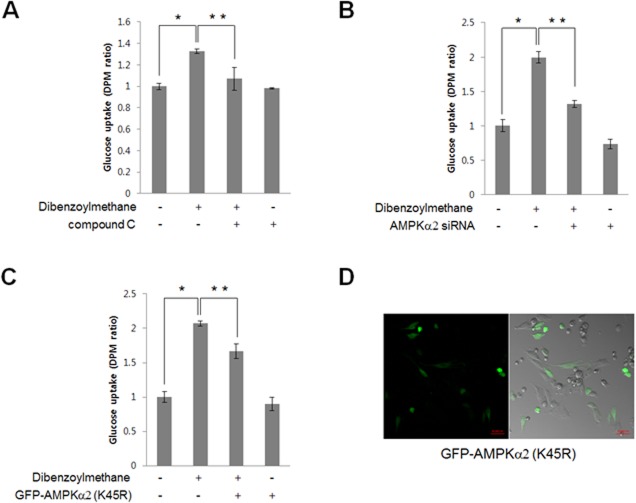
(A) L6 myotubes were differentiated for 7 days and then treated with 30 μM DBM for 1 h either in the presence or absence of compound C (1 μM). 2-deoxy-D [H^3^] glucose (2-DG) uptake was then assayed, as described in the Methods. *p < 0.05, compared with control. **p < 0.05, compared with DBM-treated cells. (B) L6 myotubes were differentiated and then transiently transfected with 50 nM AMPKα2 siRNA for 48 h. The cells were then stimulated with 30 μM DBM for 18 hours, and 2-DG uptake was assayed. *p < 0.05, compared with control. **p < 0.05, compared with DBM-treated cells. This result is representative of four independent experiments. (C) L6 myotubes were differentiated and then transiently transfected with green fluorescent protein (GFP)-AMPKα2 K45R plasmid for 48 h. The cells were then stimulated with 30 μM DBM for 18 hours, and 2-DG uptake was measured. *p < 0.05, compared with control. **p < 0.05, compared with DBM-treated cells. This result is representative of four independent experiments. D, L6 myotubes were transiently transfected with GFP-AMPKα2 K45R plasmid for 48 h. The green fluorescent signal was detected using confocal microscopy.

Among skeletal muscle cells, L6 myotubes exhibited a greater degree of glucose uptake than C2C12 cells, indicating that L6 myotubes might be appropriate model for investigating glucose uptake [25]. Thus, we examined the effect of DBM treatment on 2-DG uptake in differentiated L6 cells. DBM increased 2-DG uptake (Fig. 2A). Pre-treatment with 2 μM of compound C, an AMPK inhibitor, blocked DBM-induced 2-DG uptake, suggesting that AMPK plays a role in DBM-induced glucose uptake. To confirm that the effects of DBM were mediated by AMPK, we investigated the effects of AMPKα2 knockdown on glucose uptake. Transient transfection with AMPKα2 short interfering RNA (siRNA) was performed, and knockdown was confirmed by western blot with an anti-AMPK antibody. DBM-induced glucose uptake was not observed in cells transfected with AMPKα2 siRNA (Fig. 2B). The AMPKα2 K45R kinase dead mutant blocked the DBM effect (Fig. 2C). As shown in Fig. 2D, approximately 30–40% of cells were transfected with a green fluorescent protein (GFP)-tagged AMPKα2 K45R mutant. These results indicate that AMPKα2 was involved in DBM-induced glucose uptake.

### DBM increased AMPK phosphorylation via intracellular calcium release

**Fig 3:**
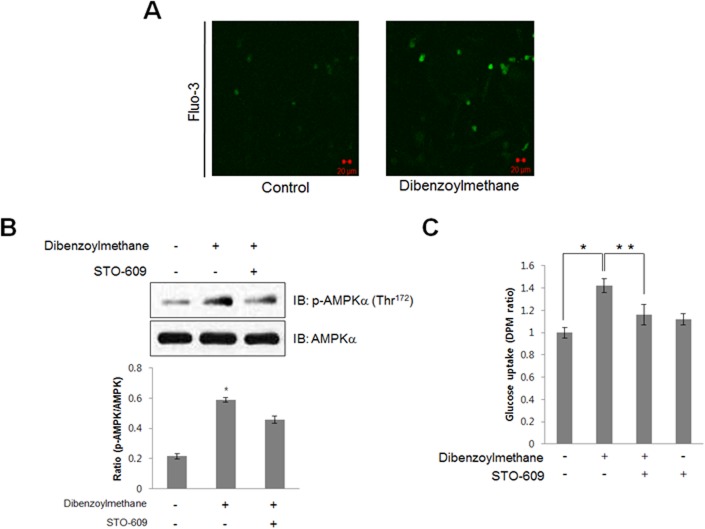
(A) C2C12 muscle cells pre-treated with Fluo-3, AM for 30 min were then treated with DBM (30 μM). The green fluorescent signal was detected using confocal microscopy. (B) C2C12 cells pre-treated with STO-609, a CaMKK inhibitor, for 30 min and then treated with 30 μM DBM for 1 h. The cells were then lysed with lysis buffer, and the phosphorylation of AMPKα2 was assessed by western blot using phosphorylation-specific antibodies. The level of total AMPKα2 was also assessed as a control for protein loading. * p < 0.05, as compared with basal condition. (C) Myoblast L6 cells were differentiated for 7 days and then pre-treated with STO-609 (1 μM) and then DBM (30 μM) for 1 h. Glucose uptake was then assayed for 2-DG uptake as described in the Methods. *p < 0.05, compared with control. **p < 0.05, compared with DBM-treated cells.

To further characterize the upstream components of the AMPK pathway, intracellular calcium levels were measured. To measure intracellular calcium, the fluorescence of the calcium-binding dye, fluo-3 AM, was measured. DBM increased fluorescence intensity in L6 cells (Fig. 3A), indicating that intracellular calcium concentration was increased by treatment with DBM. These results suggested CaMKK as a candidate for upstream signaling of AMPK, since CaMKK is activated by Ca^2+^/calmodulin binding. To test this hypothesis, C2C12 cells were pre-treated with STO-609, a CaMKK inhibitor, prior to the addition of DBM. STO-609 blocked DBM-induced AMPK phosphorylation (Fig. 3B). Moreover, pre-treatment with STO-609 blocked DBM-induced 2-DG uptake (Fig. 3C), confirming that DBM increased glucose uptake via a calcium-mediated CaMKK/AMPK pathway.

### DBM increased p38 mitogen-associated protein kinase (MAPK) phosphorylation in an AMPK-dependent manner

**Fig 4:**
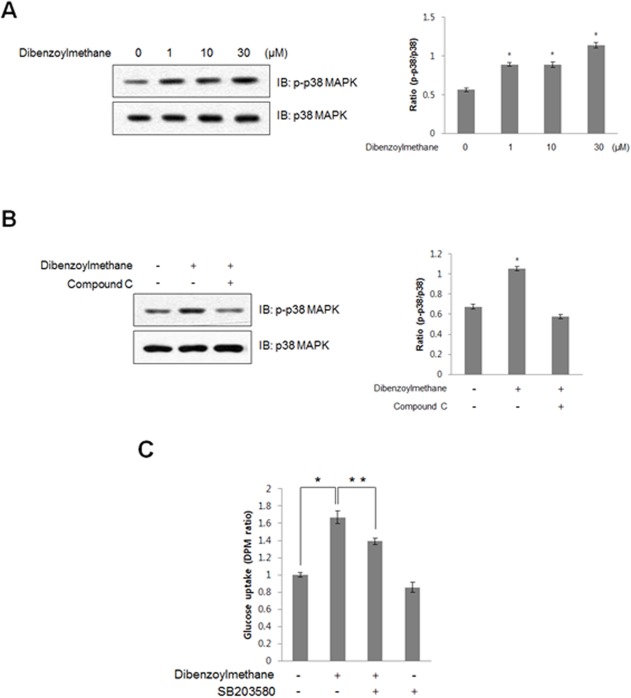
(A) C2C12 cells were stimulated with different concentrations of DBM for 1 h. The cells were lysed with lysis sample buffer, and the phosphorylation of p38 MAPK was evaluated by western blot using antibodies specific for the phosphorylated protein. The levels of total p38 MAPK were also assessed as a control for protein loading. * p < 0.05, as compared with basal condition. The results are representative of four independent experiments. (B) C2C12 cells were stimulated with 30 μM DBM for 1 h in the presence of compound C (1 μM). The cells were lysed with lysis buffer, and the phosphorylation of p38 MAPK was evaluated by western blot using phosphorylation-specific antibody. The levels of total p38 MAPK were also assessed as a control for protein loading. * p < 0.05, as compared with basal condition. Data are representative of four independent experiments. (C) Myoblast L6 cells were differentiated for 7 days and then incubated with the p38 MAPK inhibitor, SB203580, for 20 min, before cells were incubated with DBM for 18 hours. 2-DG uptake was then measured. *p < 0.05, compared with control. **p < 0.05, compared with DBM-treated cells. This result is representative of four independent experiments.

To determine which signaling pathway was downstream of AMPK, the effects of DBM on phosphorylation of p38 MAPK were examined. DBM treatment resulted in a concentration-dependent increase in p38 MAPK phosphorylation in C2C12 cells (Fig. 4A). Compound C, an AMPK inhibitor, blocked the DBM-induced increase in p38 MAPK phosphorylation (Fig. 4B). In addition, the DBM-induced increase in 2-DG uptake was abolished by treatment with SB203580, a p38 MAPK inhibitor (Fig. 4C). These results demonstrated that DBM stimulated glucose uptake via AMPK-dependent p38 MAPK activation.

### DBM increased the expression of GLUT4 and stimulated GLUT4 translocation

**Fig 5:**
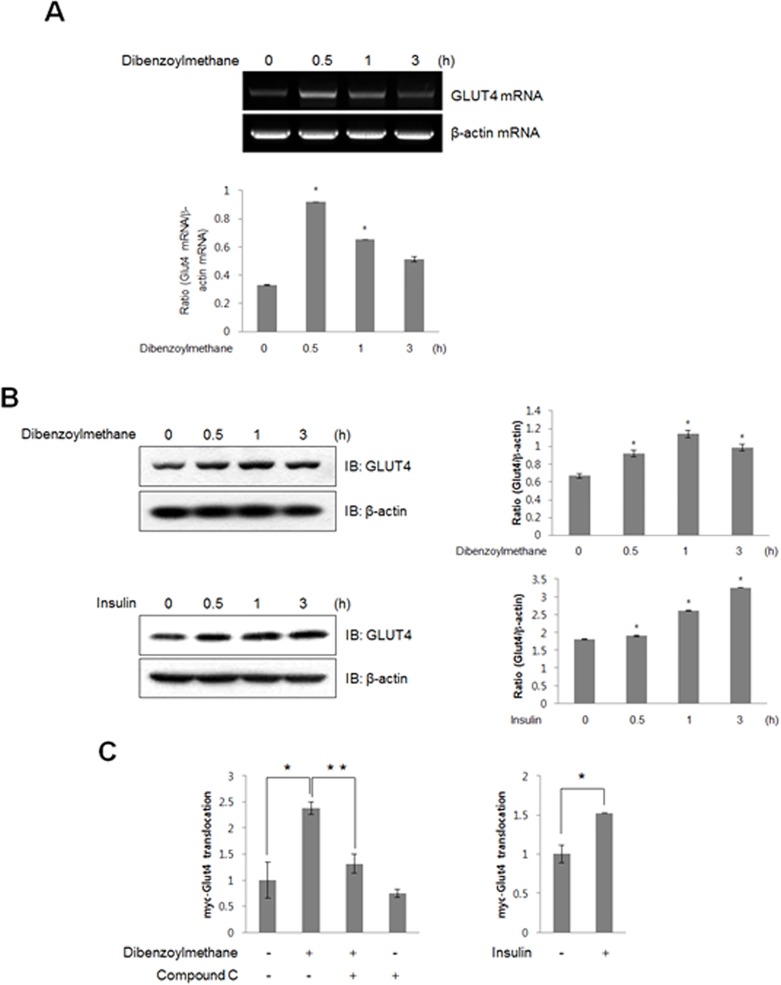
(A) Total mRNA was prepared from DBM-treated C2C12 cells, and RT-PCR was conducted using specific GLUT4 primers. The PCR products were separated on 1% agarose gels and visualized under ultraviolet light. Beta-actin was used as a positive control. * p < 0.05, as compared with basal condition. (B) C2C12 cells were stimulated for various periods with 30 μM DBM and 100 nM insulin. The cells were lysed with lysis buffer, and the expression of GLUT4 was evaluated by western blot. The levels of β-actin were also measured as a control for protein loading. * p < 0.05, as compared with basal condition. The results are representative of three independent experiments. (C) Myoblasts stably expressing L6-GLUT4myc were differentiated for 7 days and then pre-treated with compound C (2 μM), then incubated with DBM for 60 min. Insulin was treated for 30 minutes. Cell surface expression of GLUT4myc was detected using an antibody-coupled colorimetric absorbance assay. *p < 0.05, compared with control. **p < 0.05, compared with DBM-treated cells.

Based on the finding that DBM mediated glucose uptake by activating p38 MAPK, the effect of DBM on the expression of GLUT4, a downstream target of p38 MAPK, was evaluated. DBM increased the levels of GLUT4 mRNA (Fig. 5A) and protein (Fig. 5B) in C2C12 cells. The effect of insulin on GLUT4 was also performed. Colorimetric assay was used to measure cell surface localization of GLUT4myc. An increase in plasma membrane GLUT4myc was observed in the presence of DBM, indicating that DBM stimulates translocation of GLUT4 from the cytosol to the plasma membrane. This increase was not observed when cells were pre-treated with compound C (Fig. 5C). Insulin was used as positive control for GLUT4 translocation. These results demonstrated that DBM regulates glucose uptake via induction of GLUT4 expression and stimulation of GLUT4 translocation.

### DBM decreased body weight and fat accumulation in a high fat diet-induced obesity model

**Fig 6:**
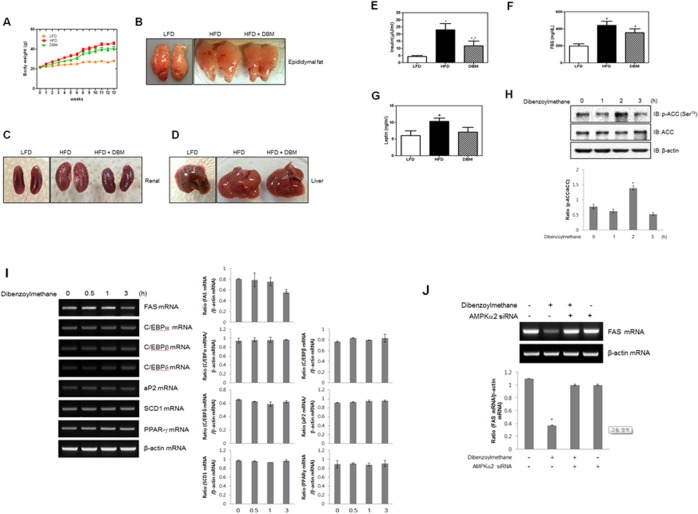
(A) Effects of DBM on body weight in an HFD-induced obesity model. The three groups were as follows: LFD, low-fat (standard) diet; HFD, high-fat diet; and HFD supplemented with 100 mg/kg/d DBM. (B) Effects of DBM on epididymal fat in an HFD-induced obesity model. (C) Effects of DBM on peri-renal fat in an HFD-induced obesity model. (D) Effects of DBM on fatty liver in an HFD-induced obesity model. (E) Effects of DBM on insulin level of HFD-induced animals. * p < 0.05, as compared with basal condition. _*_ + p < 0.05, as compared with HFD condition (F) Effects of DBM on fasting blood glucose (FBG) level of HFD-induced animals. * p < 0.05, as compared with basal condition. (G) Effects of DBM on leptin level of HFD-induced animals. * p < 0.05, as compared with basal condition. (H) 3T3-L1 pre-adipocyte cells were stimulated for indicated times with DBM. The cells were then lysed with lysis buffer, and the phosphorylation of acetyl CoA carboxylase (ACC) was assessed by western blot using antibodies specific for the phosphorylate protein. The levels of ACC were also assessed. The levels of β-actin were also measured as a control for protein loading. * p < 0.05, as compared with basal condition. The results are representative of three independent experiments. (I) Total mRNA was prepared from DBM-treated 3T3-L1 cells, and RT-PCR was conducted using specific indicated primers. The PCR products were then separated on 1% agarose gels and visualized under ultraviolet light. Beta-actin was used as a loading control. * p < 0.05, as compared with basal condition. (J) ST3-L1 cells were transiently transfected with AMPKα2 siRNA for 48 h. The cells were then stimulated with 30 μM DBM for 1 h. Total mRNA was prepared from DBM-treated 3T3-L1 cells and RT-PCR was conducted using specific fatty acid synthase (FAS) primers. The PCR products were then separated on 1% agarose gels and visualized under ultraviolet light. Beta-actin was used as a loading control. * p < 0.05, as compared with basal condition.

To determine whether DBM exerts metabolic effects in vivo, we evaluated its effects on HFD-induced changes in body weight. The administration of DBM suppressed HFD-induced increases in body weight (Fig. 6A). To observe the effect by DBM on lipid deposition, images of abdominal fat were also captured in HFD-fed animals. The amounts of epididymal and peri-renal fat were significantly lower in DBM-fed mice than in HFD-fed mice (Fig. 6B and C). The degree of fat deposition in liver due to the HFD was suppressed by DBM administration (Fig. 6D). The color, swelling, size, and fatty surface area of livers in the DBM treatment group were improved compared to controls. The images of organs of low fat diet-animals were also provided. We also analyzed blood samples of DBM-administrated mice. The increased level of insulin in HFD animals down regulated in DBM-administrated mice (Fig. 6E). Blood glucose concentrations were slightly lower in DBM–fed mice than in HFD-fed mice (Fig. 6F). Leptin level was not significantly changed by DBM administration (Fig. 6G). To explore the anti-adipogenic effect of DBM in vitro, we assessed the phosphorylation of acetyl-CoA carboxylase (ACC), a key regulatory enzyme in fatty acid synthesis, in pre-adipocyte 3T3-L1 cells. The administration of DBM increased ACC phosphorylation (Fig. 6H). Phosphorylation of ACC is indicative of a decrease in ACC activity, suggesting that DBM has anti-adipogenic activity through this mechanism. To gain additional insight into the mechanism of DBM, we used reverse transcription-polymerase chain reaction (RT-PCR) to measure the expression of various adipogenesis-related genes. Among tested genes, FAS expression was suppressed dramatically by DBM treatment (Fig. 6I). The downregulation of FAS due to DBM was not observed in AMPKα2 knockdown conditions (Fig. 6J), indicating that DBM suppresses the adipogenic process through AMPK. These results suggested that DBM played an anti-adipogenic role via AMPK-mediated adipogenic gene suppression.

## Discussion

The key finding of the present study was that DBM, a structural analogue of curcumin, exerts metabolic effects in skeletal muscle cells and pre-adipocytes via AMPK-mediated pathways, such as p38MAPK in muscle and adipogenic FAS suppression in pre-adipocytes. These findings suggest that the hypoglycemic effect of the curcumin analogue, DBM, may be attributable to regulation of AMPK activity.

Curcumin regulates glucose homeostasis in skeletal muscles via multiple mechanisms. A hypoglycemic role for curcumin was reported in an animal model of streptozocin-induced diabetes [26]. In addition, our group reported that curcumin stimulates glucose uptake in skeletal muscle cells via AMPK [18]. Even though the clinical usefulness of curcumin has been suggested, curcumin has not been used as a therapeutic agent due to its poor water solubility [27] and low bioavailability [28]. Negligible amounts of curcumin have been detected in the blood after oral administration of 1 g/kg curcumin in in vivo experiments [26]. Bioavailability of curcumin was decreased by its metabolism in vivo. For example, 99% of curcumin in plasma was detected as a glucuronide conjugate [29].

Several methods have been proposed to solve these problems, such as the use of adjuvants, nanoparticles, and liposomes. Unfortunately, these methods have not been successful thus far. The basic structural motif of curcumin has biological activity; therefore, structural modifications including the use of analogues may be promising methods to overcome solubility and bioavailability problems. In the present study, we identified DBM, a structural analogue of curcumin, for potential development as a treatment for metabolic syndromes, such as obesity and diabetes. The principal finding of this study was that DBM, a novel structural analogue of curcumin, contains the primary structural motif affects glucose and lipid metabolism.

It was recently reported that DBM, a beta-diketone analogue of curcumin, exhibits various biological activities. Although only a few comparative studies have been performed with curcumin and DBM, structural variations can often lead to differences in pharmacological activity. One study showed that the anti-tumor activity of DBM was more potent than that of curcumin [30]. With respect to metabolic function, we did not compare the metabolic potency of DBM and curcumin in skeletal muscle or pre-adipocytes, so we could not conclude which would be more promising for treating metabolic diseases. In the present study, DBM had a prominent effect on blood glucose and lipid deposition in an HFD-induced obese animal model. Therefore, our experimental results indicate that DBM may be the more useful target. More extensive studies on structure-function relationships should be performed in the future to confirm this. Another way to increase clinical utility is to develop structural analogues of DBM, which may alter its pharmacokinetics to make it more easily absorbable in the intestine or more readily metabolized to a more stable form.

Biguanides have been used to treat hyperglycemia, but their use is limited due to their side effects on the kidneys [31] and heart [32]. Medicinal plants provide the advantage of exerting pharmacological activity with few side effects. Metformin, a synthetic drug, was discovered through research on the active components of the plant Galega officinalis. Metformin, is the only approved drug for diabetes that arose from plant-based research. However, many other medicinal plants may be useful sources for the development of potential diabetes drugs.

Curcumin and its analogues are increasingly recognized as potential novel therapeutics for diabetes. In the present study, we found that DBM exerted a glucose regulatory role via the AMPK pathway in skeletal muscle cells and had an anti-adipogenic role via suppression of an adipogenic transcription factor in pre-adipocytes. In vivo, DBM blocked HFD-induced weight gain and hyperglycemia. DBM also blocked HFD-induced fat accumulation in the liver, and epididymal and peri-renal fat deposits. In a skeletal muscle cell line, p38 MAPK was associated with DBM-induced 2-DG uptake via AMPK activation. In adipocytes, FAS was found to be involved in regulating the anti-adipogenic effects of DBM. Together, these findings provide insight into the beneficial metabolic functions of DBM in skeletal muscle cells and adipocytes. These results thus suggest that DBM is a promising agent for the treatment of metabolic disorders, such as obesity and diabetes.
